# The Usefulness of Serum CXCR3 Ligands for Evaluating the Early Treatment Response in Tuberculosis: A Longitudinal Cohort Study

**DOI:** 10.1097/MD.0000000000003575

**Published:** 2016-04-29

**Authors:** Wou Young Chung, Dukyong Yoon, Keu Sung Lee, Yun Jung Jung, Young Sun Kim, Seung Soo Sheen, Kwang Joo Park

**Affiliations:** From the Department of Pulmonary and Critical Care Medicine (WYC, KSL, YJJ, YSK, SSS, KJP); and Department of Biomedical Informatics, Ajou University School of Medicine, Suwon, South Korea (DY).

## Abstract

Cell-mediated immunity plays an important role in the pathobiology of tuberculosis (TB). The ligands for CXC chemokine receptor 3 (CXCR3) activate the T-helper type 1 lymphocyte pathway. The CXCR3 ligands are reportedly useful clinical markers for the diagnosis and follow-up of TB. The objective of this study was to assess the utility of CXCR3 ligands for evaluating early treatment responses in TB.

We recruited 88 patients who underwent antituberculous chemotherapy. The serum levels of interferon (IFN)-γ and the CXCR3 ligands CXCL9 (monokine induced by IFN-γ [MIG]), CXCL10 (IFN-γ-inducible 10-kDa protein [IP-10]), and CXCL11 (IFN-inducible T-cell α chemoattractant [I-TAC]) were measured before and 2 months after the start of treatment. Treatment responses were divided into “fast” and “slow” based on the clinical, radiological, and bacteriological improvement at 2 months. A change in level of 20% or more at 2 months was defined as “significant.”

In patients with treatment success, 58 patients exhibited a fast response and 20 patients exhibited a slow response. Treatment failure occurred in 5 patients, and the diagnoses were changed to non-TB diseases in 5 patients. The levels of all CXCR3 ligands significantly decreased in the fast-response group (*P* < 0.01) but did not decrease in the other groups. IFN-γ levels showed no significant changes. The ability of significant decreases in marker levels to predict a fast response was evaluated. CXCL9 showed a sensitivity of 83%, and CXCL10 showed a specificity of 100%. Use of various combinations of CXCR3 ligands resulted in improvements in sensitivity (88%–93%), while specificity (92%–96%) was similar to that using single CXCR3 ligands. The decreases in CXCR3 ligand levels were less marked in the 2-month *Mycobacterium tuberculosis* culture-positive group than in the culture-negative group. There were significant differences in treatment outcomes in terms of 2-month culture positivity (*P* < 0.001), the significance of CXCL9 decreases (*P* *<* 0.01), and the significance of CXCL11 decreases (*P* < 0.05).

In conclusion, CXCR3 ligands may be useful surrogate markers for the evaluation of early treatment response and showed utility as indicators of possible treatment failure in TB.

## INTRODUCTION

Surrogate biomarkers are useful not only for diagnosing tuberculosis (TB) but also for treatment monitoring.^1^ Culture of *Mycobacterium tuberculosis (M. tb)*, the definitive marker of TB, has been used to determine the treatment response.^2^ However, culturing takes several weeks, and the ability of this method to predict outcomes requires improvement.^3^ A negative *M. tb* culture result in an active TB patient also compromises its utility, especially with regard to extrapulmonary TB. To overcome these shortcomings, numerous biomarkers using mycobacterial and host factors have been evaluated.^2,4–6^ Although most of the markers do not have high levels of validity, the interferon (IFN)-γ release assay (IGRA) has shown utility for the diagnosis of active TB and latent TB infection, significantly outperforming the classical tuberculin skin test.^7,8^ However, in terms of treatment monitoring, the IGRA is less useful. This shortcoming is expected, given that the IGRA is based on the TB antigen-stimulated immunologic reaction, which tends to persist despite the clinical improvement in TB.^9,10^ Unstimulated blood markers can better reflect acute changes during the early bactericidal period, but their utility is limited due to the low concentrations and lack of specificity for *M*. *tb*.^11^

Among the many mediators related to TB, chemokines in the cellular immune pathway may provide markers of TB. The CXCR3 system, closely related to IFN-γ, plays an important role in cellular immunity to TB.^12,13^ CXCR3 ligands include 3 chemokines: CXCL9 (monokine induced by IFN-γ [MIG]), CXCL10 (IFN-γ-inducible 10-kDa protein [IP-10]), and CXCL11 (IFN-inducible T-cell α chemoattractant [I-TAC]).^12^ We first reported the importance of the CXCR3 ligand release assay in TB antigen-stimulated blood.^13^ Subsequently, the blood levels of CXCR3 ligands were shown to aid in the diagnosis of TB, especially for differentiating active TB from latent TB infection.^14^ However, the diagnostic utility of CXCR3 ligand blood levels is limited, because CXCR3 ligand levels are elevated by other infections or inflammatory diseases. Serial measurements of CXCR3 ligand levels resulted in the identification of important changes during the early months and throughout the treatment period.^15^

This study focused on changes in CXCR3 ligand levels during the early months of treatment, when the markers are more important due to unavailability of *M. tb* culture and drug susceptibility testing results. Additionally, a greater number of patients underwent follow-up than in the previous study, including those with extrapulmonary TB and drug-resistant TB and those whose treatment had failed. We evaluated all 3 ligands in this study, whereas the previous study evaluated only CXCL9 and CXCL11.^15^ Therefore, we aimed to evaluate the utility of simple blood levels of CXCR3 ligands during the first 2 months of treatment.

## METHODS

### Participants

Adult patients (≥18 years of age) who were initially diagnosed with active TB and were treated with anti-TB medication for at least 2 months at Ajou University Hospital were consecutively enrolled from July, 2013 to September, 2015.

The study was approved by the Ajou University Hospital Institutional Review Board. All subjects provided written informed consent.

### Evaluation and Treatment of TB

A diagnosis of active TB was made when *M. tb* was identified in clinical specimen cultures or, in the case of negative culture results, when the clinical manifestations, laboratory findings, pathologic evaluation, and radiographic findings were compatible with TB and were supported by an adequate response to anti-TB treatment. The cases were reviewed independently by 2 pulmonary medicine physicians and 1 radiology specialist.

The evaluation and treatment of TB were based on the Korean Guidelines for Tuberculosis 2011.^16^ The clinical samples were stained for acid-fast bacilli and cultured for mycobacteria. Treatment was initiated using a standard regimen of rifampicin, isoniazid, pyrazinamide, and ethambutol. The minimum duration of therapy was 6 months.

### Blood Sampling

Baseline blood samples were obtained from the TB patients before treatment and follow-up blood samples were obtained 2 months after the start of treatment. Sera were prepared after centrifugation at 1500 × *g* for 10 minutes and then immediately frozen at − 80 °C.

### Measurement of Serum CXCR3 Ligands and IFN-γ Levels

The serum levels of CXCL9, CXCL10, CXCL11, and IFN-γ were measured using Human Quantikine enzyme-linked immunosorbent assay kits (R&D Systems, Inc., Minneapolis, MN).

### Definitions of Diagnosis, Treatment Response, and Changes in Marker Levels

The treatment was considered a “failure” when sputum smear or culture was positive and/or clinical findings were not improved at 5 months or later.^17^ The treatment responses of the treatment-success group were dichotomized according to clinical status at 2 months. The response was defined as “fast” when all 3 of the following criteria were fulfilled within 2 months: negative smear and culture for acid-fast bacilli; improvement in TB-specific symptoms, including fever, cough, weight loss, and/or night sweats; and improvement in clinical and radiographic lesions previously identified as consistent with TB. If any of the factors persisted after 2 months, the treatment response was considered “slow.”

With respect to the changes in marker levels, a change of ≥20% from the baseline levels at 2 months was defined as “significant.”

### Statistical Analysis

All statistical analyses were performed using SPSS ver. 22 (SPSS Inc., Chicago, IL). Numerical data were expressed as medians (interquartile range). The Mann–Whitney *U* test and Kruskal–Wallis test were performed to compare groups. The Chi-square test with Fisher exact test was employed to compare binary variables. A Wilcoxon paired test was performed to evaluate the significance of changes in a group.

To evaluate the performance of the markers, the sensitivity, specificity, positive predictive value (PPV), negative predictive value (NPV), and F-score were calculated using conventional formulae. F-score is a measure of test accuracy that considers both precision and recall by calculating the harmonic mean of PPV and sensitivity.^18^ Performance measures were also calculated using various combinations of the markers. A *P-*value of <0.05 was deemed to indicate statistical significance.

## RESULTS

### Clinical Characteristics of the Study Population

The demographic characteristics of the study subjects are presented in Table 1. Of the 5 patients whose treatment failed, 3 had multidrug resistant TB, and 1 had resistance to only isoniazid. The remaining patient had cervical lymphadenitis, and drug susceptibility testing was not performed due to a negative *M. tb* culture. The patients with treatment failure were subsequently treated with secondary TB medications. One patient with multidrug resistant TB died. The treatment duration of 9 patients in the treatment-success group was extended to 9 to 12 months according to the physician's decision based on the clinical and radiological status after 6 months of treatment. The slow-response group showed a significantly higher incidence of treatment extension than the fast-response group (7/20 vs 2/58, *P* < 0.001). One of the patients with a slow response had isoniazid-resistant *M. tb*.

**TABLE 1:**
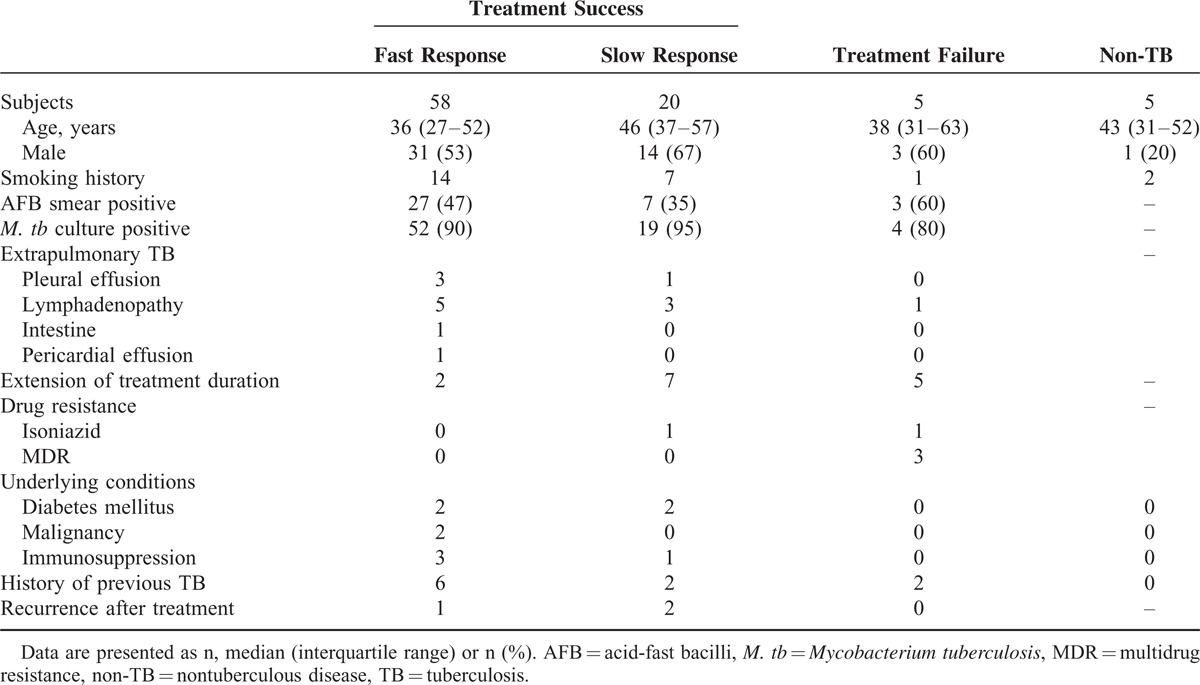
Demographic Data

The diagnosis of 5 patients was changed to non-TB diseases after 2 months of anti-TB medication. The corrected diagnoses were pulmonary fibrotic sequelae (n = 2), nontuberculous mycobacterial infection (n = 2), and pneumoconiosis (n = 1).

### Changes in Marker Levels and Treatment Response

**FIGURE 1:**
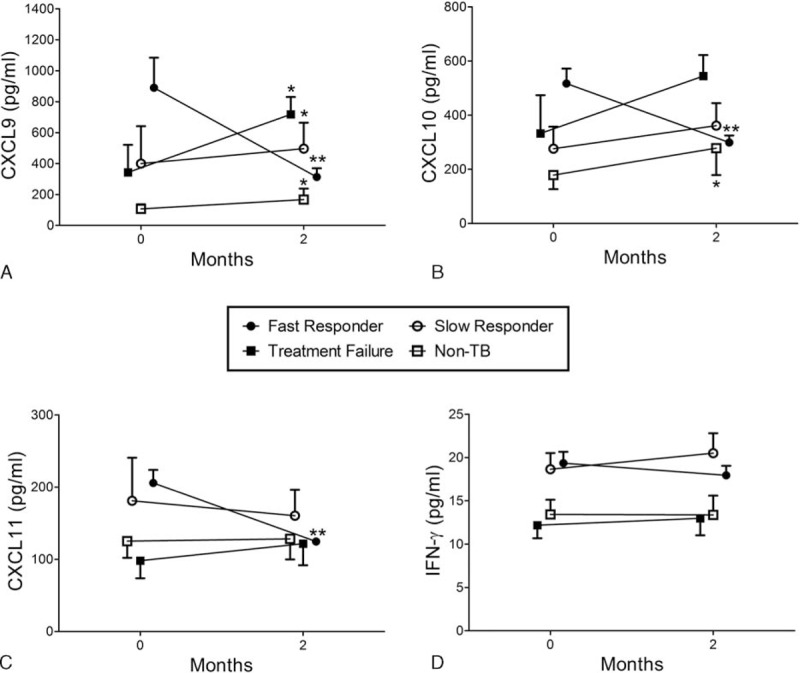
The serum levels of (A) CXCL9, (B) CXCL10, (C) CXCL11, and (D) interferon (IFN)-γ before and 2 months after the start of treatment in the following TB patient subgroups: fast responders with treatment success (●, n = 58), slow responders with treatment success (○, n = 20), TB patients with treatment failure (▪, n = 5), and non-TB patients (□, n = 5). The time points on the horizontal axis represent before treatment (baseline, 0 months) and 2 months after the initiation of treatment. The treatment response was defined as “fast” when all bacteriologic, radiologic, and clinical features of TB were improved at 2 months of treatment. If any of these features persisted after 2 months, the treatment response was considered to be “slow.” ^∗^*P* < 0.05 and ^∗∗^*P* < 0.01 compared with baseline levels.

Among those in the treatment-success group with fast responses, the levels of all CXCR3 ligands significantly decreased within 2 months (all *P* < 0.01). In the slow responders, CXCL9 levels increased, whereas those of other markers did not change significantly. In the treatment-failure group, the levels of all CXCR3 ligands increased, but only that of CXCL9 reached statistical significance (*P* = 0.04). IFN-γ levels did not show significant changes in any of the groups (Figure 1). Treatment responses were correlated with changes in CXCR3 ligand levels (Table 2). A significant decrease in the CXCL9 level was found in 84.5% of the fast-response group. In the slow-response group, no patients showed significant decreases in CXCL10 levels.

**TABLE 2:**
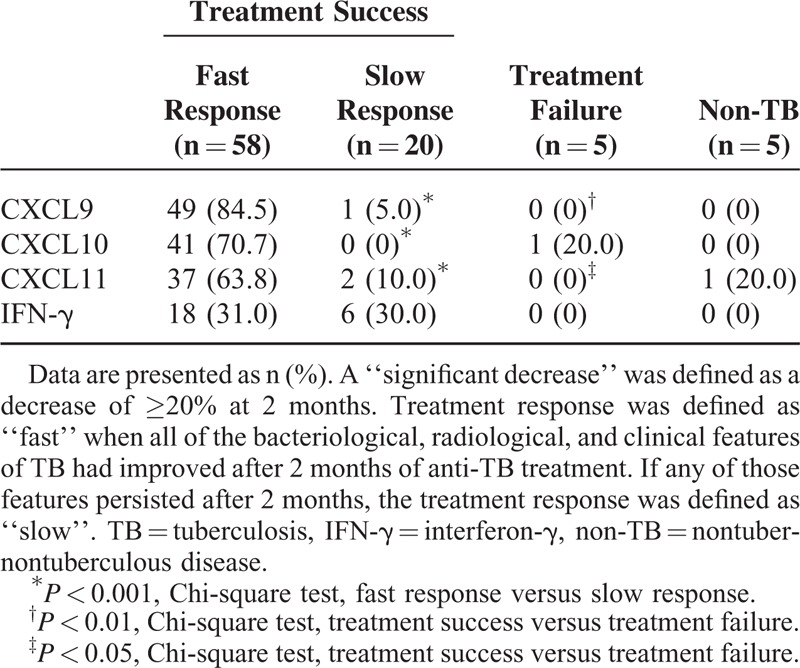
Number of TB Patients With Significant Decreases in Marker Levels by Treatment Outcome and Response

### Performance of Markers for Evaluation of Early Treatment Responses

The ability of significant decreases in the levels of single markers and of combinations thereof to predict a fast response was evaluated. Significant decreases in the levels of CXCL9/CXCL10, CXCL9/CXCL11, and CXCL10/CXCL11 combinations were defined as a significant decrease in at least 1 of the 2 markers. A significant decrease in the levels of combinations of 3 CXCR3 ligands was defined as a significant decrease in any 1 or 2 of the markers. IFN-γ was excluded because of its very low predictive performance. The sensitivity and NPVs of the CXCR3 ligands were relatively lower than their specificity and PPVs. These shortcomings were overcome by the use of combinations of 2 or 3 CXCR3 ligands. Among the 2-marker combinations, CXCL9/CXCL10 produced the best results, with a sensitivity of 0.88, an NPV of 0.77, and an F-score of 0.93. The best performance was obtained when a significant decrease in the levels of combinations of 3 CXCR3 ligands was defined as a significant decrease in any one of the markers, as evidenced by an F-score of 0.95 (Table 3).

**TABLE 3:**
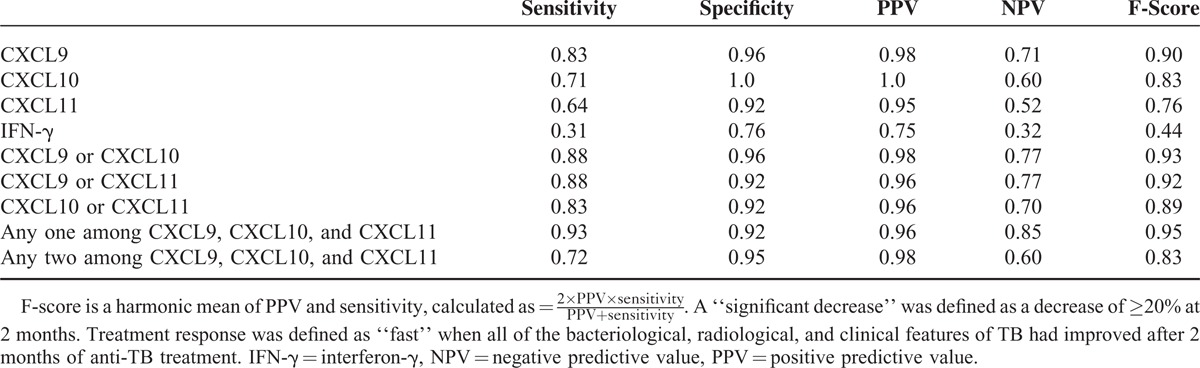
Ability of Significant Decreases in the Levels of Single Markers and Combinations Thereof to Predict a Fast Response After 2 months of Treatment

### Clinical Significance of *M. tb* Culture Results and Changes in CXCR3 Ligand Levels

Patients (n = 65) were followed up at 2 months by means of sputum *M. tb* cultures. The TB patients with negative 2-month *M. tb* culture results had greater decreases in CXCR3 ligand levels than those with positive 2-month *M. tb* culture results (Table 4). Two-month *M. tb* follow-up cultures were performed in 4 patients with treatment failure, and all showed persistently positive results. A Chi-square test showed significant differences in the treatment outcome according to 2-month *M. tb* culture positivity (Table 4). Additionally, the decreases in the CXCL9 and CXCL11 levels were significantly associated with the treatment outcome (Table 2).

**TABLE 4:**
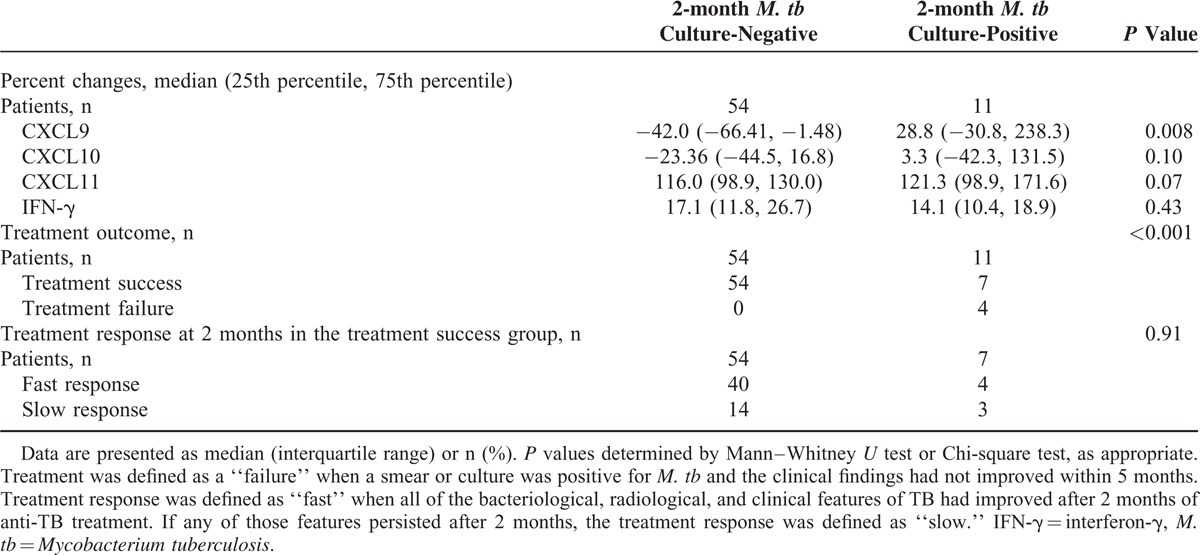
Comparison of Percentage Changes in Marker Levels, Treatment Outcomes, and Responses According to *M. tb* Culture Positivity at 2 months After Initiation of Antituberculous Medication

## DISCUSSION

We found that the changes in serum CXCR3 ligand levels during the first 2 months of anti-TB treatment were reflective of the treatment response. Of the 3 ligands, CXCL9 predicted the treatment response best. The changes in CXCR3 ligand levels were also correlated with the *M. tb* culture results at 2 months of treatment. The changes in serum IFN-γ levels were not correlated with the 2-month treatment response and were less capable of predicting a rapid treatment response compared with the changes in serum CXCR3 ligand levels.

The popular IGRA, which measures TB-antigen-stimulated IFN-γ levels, was reported to have a poor correlation with the treatment response, showing blunted level changes and fluctuations during the longitudinal monitoring of TB.^9,10^ This shortcoming is expected because cell-mediated immune reactions may persist throughout the treatment period.^19^ Furthermore, stimulation with TB antigens further blunts the changes in IFN-γ levels.

CXCR3 ligands are produced as part of the host response to TB. Once TB is controlled, the stimulus dissipates and the levels of these chemokines should decrease. The unstimulated levels of CXCR3 ligands may be appropriate for reflecting these changes. Although the unstimulated blood levels of CXCR3 ligands are not TB-specific and may be lower than the TB-antigen stimulated levels used in the IGRA methodology, they have advantages in that they reflect acute changes in the markers, and the sampling procedure is simple, convenient, and readily available. In the case of IFN-γ, however, even its unstimulated levels failed to represent the early treatment response. In previous studies, the predictive performance of serum IFN-γ levels for the diagnosis and treatment monitoring of TB was generally inferior to that of CXCR3 ligands.^13–15^ These results may be attributed to the specificity of CXCR3 ligands to the Th1 lymphocyte pathway^12,20^ and their higher concentrations compared with IFN-γ.

From our results, the lower 2-month decreases in CXCR3 ligand levels can indicate a slow treatment response but not necessarily eventual treatment failure. A slow 2-month treatment response may also occur in severe TB due to prolonged inflammatory reactions, paradoxical responses, and a lack of patient treatment adherence.^21,22^ Therefore, changes in marker levels should be considered in conjunction with the overall clinical findings. To predict treatment failure, further follow-up of serum CXCR3 ligand level changes is required, given that they continue to change throughout the treatment period.^15^

In this study, a minimum 20% decrease in chemokine levels was used to indicate a significant change based on usual practice, but a different cutoff level may also be useful. Although the 20% decrease was used as the cutoff for significance, successful treatment correlated with greater decreases. Further studies are required to define the optimal percentage for the significant changes in serum CXCR3 ligands.

The absence of significant decreases in the CXCR3 ligand levels during the initial 2 months may be a useful indicator of the need to prolong treatment. Extension of the treatment duration is usually recommended in severe and disseminated TB, cavitary TB, positive 2-month *M. tb* culture, and delayed treatment responses, although no consensus regarding absolute indications has been established.^16,23,24^ From our results, patients without significant decreases in CXCR3 ligand levels during the initial 2 months had a higher incidence of treatment extension after the standard 6 months of therapy than did those with significant decreases in CXCR3 ligand levels. However, these data are not presented, because the treatment extension data were of limited consistency. In this study, treatment extension was not determined using predefined indications but was subjectively decided by the physicians in charge.

Patients with treatment failure due to drug resistance should be identified as early as possible not only to achieve the best treatment outcome but also to control the spread of TB in society. Surrogate markers play an important role in the first 2 months of treatment, when bacteriologic information is limited. The change in serum CXCR3 ligand levels may play a supplementary role in the determination of treatment response.

From our results, serum CXCL11 levels were not as strong of an indicator of treatment response, but CXCL11 induction was better than the other 2 CXCR3 ligands for the diagnosis of TB.^13^ The in vitro potency and receptor affinity were reported to be highest for CXCL11 among the CXCR3 ligands.^25,26^ However, 2 subsequent studies evaluating unstimulated blood levels of CXCR3 ligands showed that serum CXCL11 levels were lower with inferior diagnostic performance compared with those of the other 2 CXCR3 ligands.^14,15^

Combinations of CXCR3 ligands had improved predictive performance. A previous study found that CXCR3 ligands had complementary roles in treatment monitoring.^15^ The use of combinations of CXCR3 ligands may result in better performance by a mutual complementary role, as they have different mechanisms of regulation and action.^12^ However, the combination could not produce well-balanced improvements in the sensitivity and specificity. The combination can be helpful in some situations, but its use should be decided prudently. From a practical perspective, the use of a single marker, especially CXCL9, should be considered preferentially, because it may have advantages by presenting consistently excellent results without complex manipulation of multiple marker levels.

This study had several limitations. First, our sample size was small, particularly in the treatment-failure group. Further research should involve a more heterogeneous mix of cases to validate the results. Second, as described earlier, the criteria for treatment response and for the significance of the changes in marker levels were somewhat arbitrary and subjective because no standardized universal criteria exist. Third, the study did not look at changes in the marker levels that may occur during other infections or inflammatory responses, nor did it control for these parameters. Therefore, these conditions may bias the results in the practical application of serum CXCR3 ligand levels.

## CONCLUSION

Measuring the levels of CXCR3 ligands appears to be a rapid, valid, and useful method to monitor the success of anti-TB therapy, which other methods, including *M. tb* culture and IGRA, do not provide. Therefore, they can be useful as surrogate markers of the clinical response during the initial 2 months of TB treatment and may play a role as indicators of possible treatment failure.
